# Supplementary material for: Improving Uptake and Sustainability of Sanitation Interventions in Timor-Leste: A Case Study
Source: Int J Environ Res Public Health. 2021 Jan 24;18(3):1013. doi: 10.3390/ijerph18031013 (PMC7908170; doi:10.3390/ijerph18031013)
Supplement: Supplementary file 1 [file ijerph-18-01013-s001.pdf]

International Journal of
Environmental Research and Public Health

MDPI

Supplementary Materials: Improving Uptake and Sustainability of Sanitation Interventions in Timor-Leste: A Case Study

Stakeholders were asked to indicate their level of agreement or disagreement with eight statements relating to the roll-out of interventions in communities. Responses were graded from 1–5, with 1 and 2 indicating strong disagreement and disagreement respectively (**Figure S1**, orange bars), and 4 and 5 indicating agreement and strong agreement respectively (**Figure S1**, green bars); 3 indicated a neutral response to the statement (**Figure S1**, grey bars).

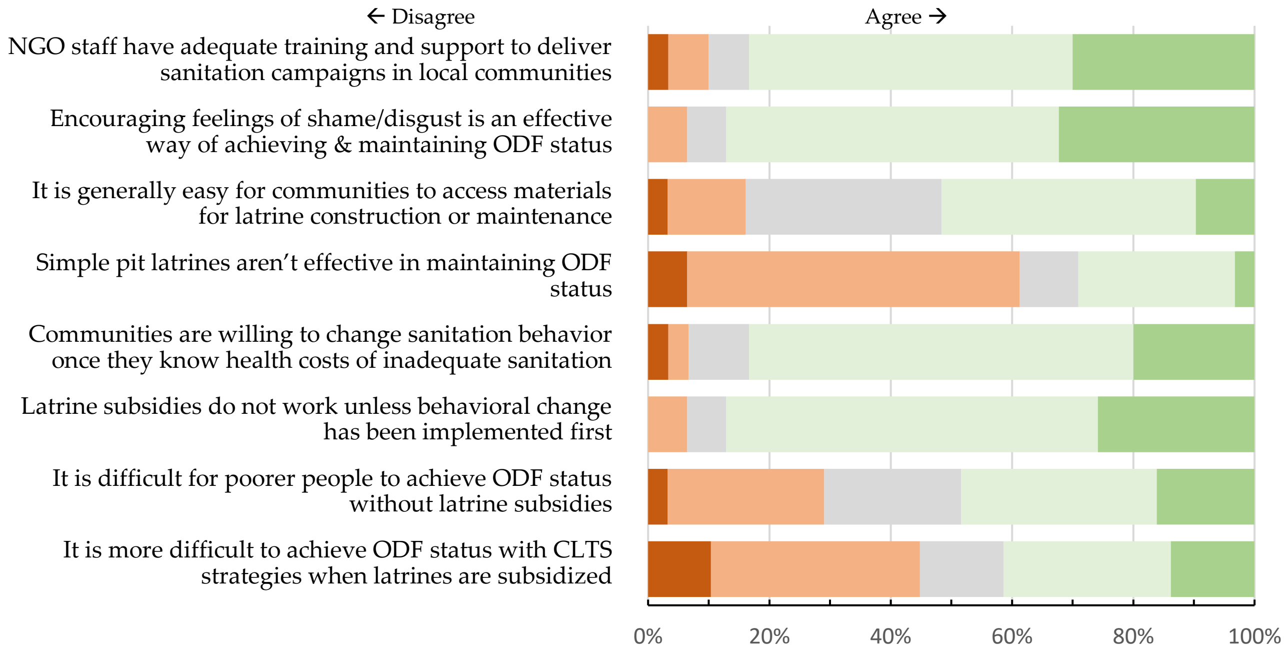

Figure S1. Stakeholder opinions in response to statements on sanitation and ODF status. Dark orange and pale orange bars represent strong disagreement and disagreement respectively; dark green and pale green bars represent strong agreement and agreement respectively; grey bars represent a neutral response.
